# Role of the telomeric factor TRF2 in post-hypoxic brain damages

**DOI:** 10.1016/j.redox.2024.103278

**Published:** 2024-07-25

**Authors:** Shuaiyun Gao, Sheng Huang, Yiwen Xu, Bo Wang, Peng Cheng, Yiming Lu, Eric Gilson, Jing Ye

**Affiliations:** a Department of Geriatrics, Medical Center on Aging of Shanghai Ruijin Hospital, Shanghai Jiaotong University School of Medicine, Shanghai, China; b Pôle Sino-Français de Recherches en Sciences du Vivant et Génomique, International Research Project Cancer, Aging and Hematology, RuiJin Hospital, Shanghai Jiao Tong University School of Medicine/CNRS/INSERM/University Côte d'Azur, Shanghai, China; c The State Key Laboratory of Medical Genomics, Shanghai, 200025, China; d Department of Emergency, Ruijin Hospital, Shanghai Jiao Tong University School of Medicine, Shanghai, 200025, China; e University Côte d'Azur, Inserm, CNRS, IRCAN, Nice, France; f Department of Medical Genetics, IHU RespirERA, CHU, Nice, France

**Keywords:** *Terf2*, Excitotoxicity-like phenotype, Neurons, Neurotransmitter, Glutamate

## Abstract

The neuronal excitotoxicity that follows reoxygenation after a hypoxic period may contribute to epilepsy, Alzheimer's disease, Parkinson's disease and various disorders that are related to inadequate supplement of oxygen in neurons. Therefore, counteracting the deleterious effects of post-hypoxic stress is an interesting strategy to treat a large spectrum of neurodegenerative diseases. Here, we show that the expression of the key telomere protecting protein Trf2 decreases in the brain of mice submitted to a post-hypoxic stress. Moreover, downregulating the expression of *Terf2 i*n hippocampal neural cells of unchallenged mice triggers an excitotoxicity-like phenotype including glutamate overexpression and behavioral alterations while overexpressing *Terf2* in hippocampal neural cells of mice subjected to a post-hypoxic treatment prevents brain damages. Moreover, *Terf2* overexpression in culture neurons counteracts the oxidative stress triggered by glutamate. Finally, we provide evidence that the effect of *Terf2* downregulation on excitotoxicity involves *Sirt3* repression leading to mitochondrial dysfunction. We propose that increasing the level of *Terf2* expression is a potential strategy to reduce post-hypoxic stress damages.

## Highlights

• The brain level of telomeric factor *Terf2* decreases upon hypoxia.
• Overexpression of *Terf2* in the hippocampus reduces the brain damages due to hypoxia, including glutamate release.
• A downregulation of *Terf2* in the hippocampus is sufficient to trigger an excitotoxicity effect.
• *Terf2* controls brain homeostasis upon hypoxia by the regulation of the expression of the mitochondrial Sirtuin SIRT3.

## Graphical abstract

**Figure:**
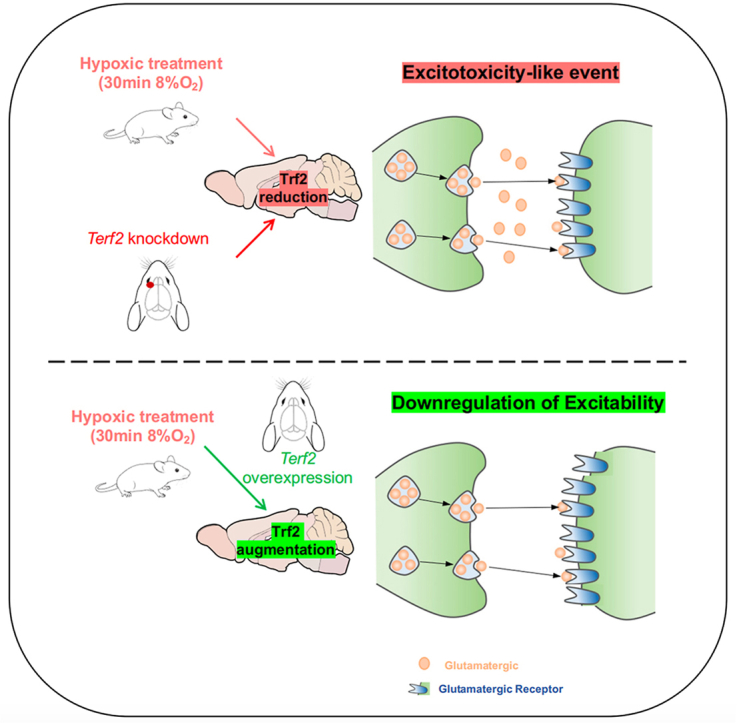

## 1 Introduction

Post-hypoxic stress elicits various damaging processes including ischemia-reperfusion, obstructive sleep apnea, pulmonary hypertension, ischemic heart failure as well as global and focal cerebral ischemia [1]. One of the main pathogenic manifestations of post-hypoxic stress in the central nervous system is neuronal loss by increasing the production of Ca^2+^ and glutamate, which triggers excitotoxicity [[2], [3], [4]].

Long-lived post-mitotic cells, such as myofibers and neurons, can live for decades in the body. Several publications have reported that in addition to their well-established role in cellular senescence of mitotic cells [5], telomeres may behave as an aging clock in long-lived post-mitotic cells [6]. First, cardiomyocytes acquire a senescent-like phenotype during aging characterized by persistent DNA damage at telomeres that is driven by mitochondrial dysfunction in a telomere length-independent manner [7]. Second, downregulation in myotubes of the *Terf2* gene encoding shelterin subunit TRF2 triggers *Sirt3* gene repression and subsequently mitochondrial dysfunction and ROS production [8]. Third, TRF2 appears to control neuronal biology through multiple mechanisms [[9], [10], [11], [12], [13], [14]]. So, we investigated the role of Trf2 in brain homoeostasis following a period of hypoxia in mice. We found that the expression of the *Terf2* gene is down-regulated during post-hypoxic stress and that its inhibition in the hippocampus led to mitochondrial dysfunction, glutamate overproduction and behavioral alterations. This led us to propose that increasing the TRF2 level in the brain is a potential strategy to counteract the damaging effects of post-hypoxic stress.

## 2 Methods

### 2.1 Stereotaxic injection

The left and right halves of the mice's brains were fastened with ear bars and localizers after anesthesia. After sterilization with 75 % ethanol, a 1 cm incision was made along the mid-suture, revealing the whole skull, followed by the meninges being digested with 3 % hydrogen peroxide. Using a localizer, the location of the hippocampal Dentate Gyrus (DG) area is determined (Bregma being the coordinate origin, −1.5 mm to the left and −1.8 mm to the back). The microinjector rod containing the aspirated lentivirus is then uniformly inserted downward (1.8 mm), and 5 μL of the lentivirus is then injected uniformly. Finally, the sutures are sterilized and the mice are kept warm until awake.

### 2.2 Hypoxia model

Two-month-old mice were given 8 % oxygen for 30 min as a hypoxia group, followed by a 30-min recovery in normoxia so they could perform in the subsequent behavioral tests [15].

### 2.3 Behavioral tests

All behavioral tests were performed in the facility from Shanghai Xinruan Animal Behavioral Recognition Technology.

*Light-Dark Box*: Directly beneath the camera, a 7.5 cm × 7.5 cm channel divides the dark field from the light field, of which the top is an open area. Within 5 min, it was noted how many times the mice were moved between the light and dark fields, how long they stayed there, and how far they traveled [16].

*Novel Object Recognition*: A black cylindrical test field was placed directly below the camera, and two objects A and B were placed at the same distance from the wall of the barrel. For 10 min, the mice were left in the field to acclimate and explore. The mice were placed back in the field for 5 min after object A was changed to object A′, which was the same color and size but had a different form, after 1 h had passed. The exploration time and distance of A, B and A′ were recorded during the two explorations. This experiment can be used to evaluate mice's short-term memory, their capacity to distinguish novel items, and their motivation to explore depending on the duration and distance of their explorations [17].

*Self-care behaviors*: Mice were placed in an open field and the latency of their hair grooming and licking behaviors were recorded using a camera for 10 min.

### 2.4 Primary neuronal cultures and treatments

Newborn mice were removed within 24 h of birth, soaked in 75 % alcohol for 5 min, and placed in cold saline under a microscope. The mouse cortex was separated and cut into 1-mm pieces. After digestion with trypsin with EDTA (Gibco) for 15 min, the mixture was filtered and the filtered liquid was centrifuged at 800 rpm. The precipitate is the primary neuronal cells. Culture with seed plate solution (Neurobasal®-A Medium (1x) + B27+Glutamic acid, Gibco and Stem Cell) and spread into PDL (Poly-d-Lysine hydrobromide, Sigma)-treated dishes prepared in advance. After overnight observation, if the cells are basally adhered, they are fully replaced with the culture medium (Neurobasal + B27 + 0.5 mM l-Glutamine).

### 2.5 Lentivirus

All lentivirus package was done in Shanghai Genomeditech. The sequences are listed in Table S4. After being swiftly warmed up from −80 °C, the calculated volume of lentivirus was added to the pre-layered antibiotic-free medium of culture cells. The entire volume of the medium was altered after 72 h of regular cultivation. Cell samples were taken 7–10 days after lentivirus infection, and RT-qPCR or Western Blot were used to determine the effectiveness.

### 2.6 qRT-PCR

The RT-qPCR primers were created in accordance with the guidelines for primer design. HiScript III RT SuperMix for qPCR (Vanzyme) was used to reverse-transcribe the total RNA after it had been extracted using the TRIzol reagent (Invitrogen). The Analytikjena qTOWER3 G (Jena) and ChamQ SYBR qPCR Master Mix (Vanzyme) were used for the RT-qPCR analysis, which included pre-denaturation at 95 C for 30 s, amplification at 95 C for 30 s and at 60 C for 10 s for 40 cycles. Using 2^△△Ct^, the relative mRNA levels were determined. All primers used are listed in Table S2.

### 2.7 Immunofluorescence and FISH

#### 2.7.1 Immunofluorescence

The slides (cell or brain tissue) were rewarmed to room temperature, submerged in 4 % PFA for roughly 15–20 min, then put on a horizontal shaker and rinsed three times with 0.3 % Trition X-100 (in PBS) solution for 10 min each time. They were then blocked for an hour at room temperature with 5 % BSA +0.5 % Trition X-100 (in PBS). Primary antibody diluted in the blocking buffer were added dropwise and incubated overnight in 4 °C. The leftover primary antibody was eliminated the next day by shaking the same 0.3 % Trition X-100 (in PBS) solution three times for a total of 10 min. After that, the secondary antibody was diluted in blocking solution and applied dropwise before being incubated at 37 °C for an hour. The slices were sealed in Vectashield (Vector) and stored in damp boxes at 4 °C after being shaken and washed in the same manner, stained in DAPI for 5 min, shaken and washed in PBS for 10 min. All antibodies used are listed in Table S3.

#### 2.7.2 FISH

The slides were rewarmed to room temperature, submerged in 4 % PFA (Sangon) for roughly 15–20 min, then put on a horizontal shaker and rinsed three times with 0.3 % Trition X-100 (in PBS) solution for 10 min each time. The slides were then sequentially soaked in 75 %, 95 % and 100 % ethanol for 5 min each to be fully dehydrated. The probe diluted 1:100 was then applied to the sample surface. The samples were denatured at 85 °C for 5 min, and thereafter placed in a dark and wet box for 2 h at 37 °C for hybridization. Being immersed and shaken in Wash I and Wash II solutions (recipes showed in Table S1) twice for 15 min each, the probe was then microscopically inspected (Leica) for the next step, such as antibody incubation. At last, A2 and AXIO Observer Z1 and LSM 880 microscopes were used for image acquisition. Fig. S1A shows the observation sites of mouse brain tissue [18].

### 2.8 SA-b-gal

Senescence β-galactosidase Staining Kit (Beyotime) was used to stain the slides in accordance with the manufacturer's instructions. Fig. S1A shows the observation sites of mouse brain tissue [16].

### 2.9 TdT-mediated dUTP Nick-End Labeling (TUNEL)

TUNEL was performed according to the manufacturer's protocols of In Situ Cell Death Kit (Roche). Fig. S1A shows the observation sites of mouse brain tissue [16].

### 2.10 Metabolomic analysis

#### 2.10.1 Sample pre-process

Weigh 50 mg brain sample in a centrifuge tube. Add 400 μL 50 % methanol and magnetic beads to break. 25000 rpm, centrifuge for 2min for use. Preparation of standard song: Take 39 kinds of neurotransmitter standard products and mix Solution, carry out gradient dilution (CAL1-CAL9). Take 20 μL sample and standard song, add 60 μL pre-cooled 50 % methanol, shake for 5min, precipitate at −20 °C for 4h, centrifuge at 20000g at 4 °C for 15min, take 20 μL of supernatant in EP Tube. Derivatization: Add 20 μL 200 mM 3-NPH and 20 μL 120 mM EDC-6% Pyridine mixture to the EP tube containing 20 μL supernatant, incubate with shaking at 25 °C for 30min, centrifuge, take the supernatant, and carry out LC-MS/MS analysis.

### 2.11 LC-MS/MS detection

The analytical instrument of this project is Waters Iclass-AB Sciex 6500 liquid-mass tandem mass spectrometry system, and the chromatographic column is waters BEH C18 (model: 1.7 μm*2.1*100 mm).

Metabolomics analysis was performed by *Beijing Genomics institution*.

### 2.12 Statistical analysis

Analysis was performed using Prism 9, and the analysis methods included Paired *t*-test and One-way ANOVA analysis (*: p < 0.05; **:0.01 < *p* < 0.05; ***:0.001 < *p* < 0.01; ****: p < *0.001*).

### 2.13 Materials

All Primers and Antibodies are listed in Supplementary Tables S1–4.

### 2.14 Animals

All of the C57/B6J mice of 3 months age used in the study were bred in the facilities of Shanghai Phenotek Biotechnologies and Shanghai Ruijing Hospital, Shanghai Jiaotong University School of Medicine, Laboratory in Hematology and Cancer. Mice were kept no more than 5/cage. All animal procedures were approved by the Animal Care and Use Committee.

## 3 Results

### 3.1 Model of post-hypoxic brain damage

**Fig. 1:**
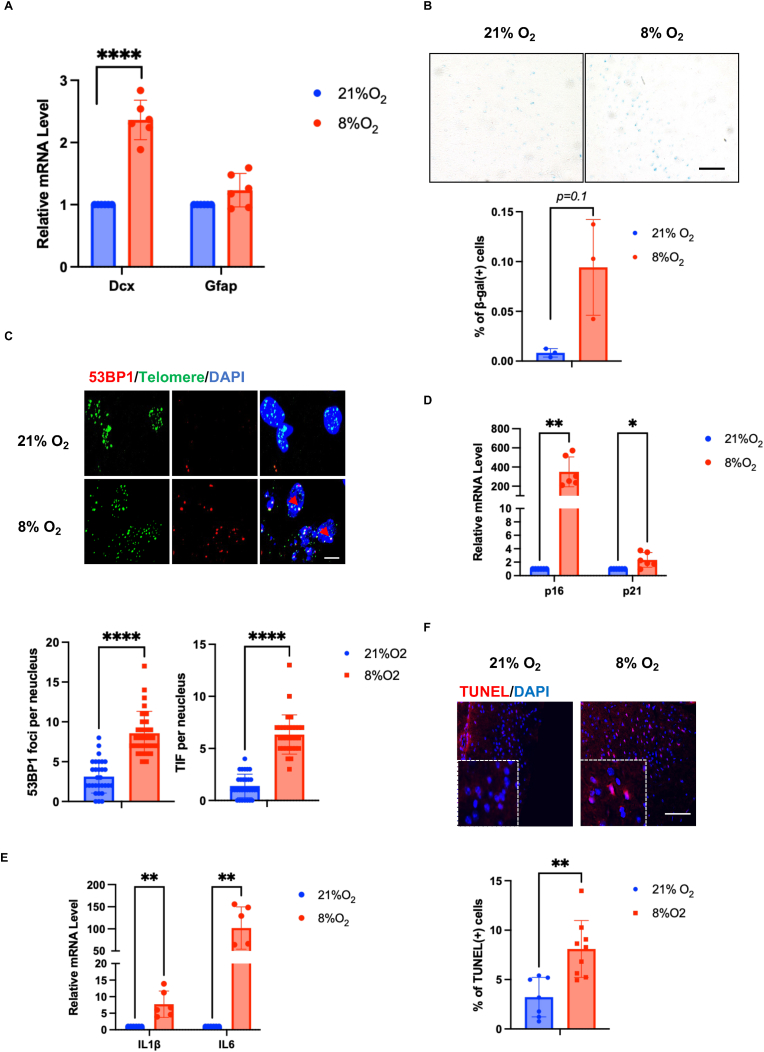
Hypoxic stress induced brain damage of 3-month-old mice. A. Expression of neuron-specific genes Dcx and GFAP upon hypoxia in mouse brain by RT-qPCR by (n = 6). B. % of β-galactosidase (β-gal) positive cells after 30-min-hypoxic treatment in mouse brain (n = 3), *Scale bar:50um*. C. Confocal imaging showed the number of 53BP1 foci (red) and TIFs (yellow, 53BP1 foci colocalized with telomere signal) in mouse brain after 30-min-hypoxic treatment (n = 3). *Scale bar:10um*. D-E. Expression of cell-cycle checkpoint factors, p16 and p21, as well as of the inflammation factors IL1β and IL6 increased in brain tissues upon hypoxia by RT-qPCR(n = 6). F. % of TUNEL positive cells in mouse brain (n = 3) *Scale bar:20um*. Paired *t*-test for A, D-F; Mann Whitney *U* test for B–C; *:p < 0.05; **:0.01<p < 0.05; ***:0.001<p < 0.01; ****:p < 0.001. (For interpretation of the references to color in this figure legend, the reader is referred to the Web version of this article.)

To create an experimental setting of post-hypoxic brain damage, wild-type mice were submitted to a 30-min treatment in a closed hypoxic chamber (8 % O_2_) [15]. After a 30 min return to normoxia, brain tissue damages appeared (Fig. S1B) as well as an increased level of neurogenesis as revealed by the newborn neuronal marker Doublecortin (Dcx), whereas the expression of the glial marker GFAP did not change significantly (Fig. 1F). As previously observed in post-ischemic situations [19], the level of several markers of cellular senescence increased: β-galactosidase (β-gal, Fig. 1A), DNA damage (53BP1,Fig. 1B), telomere damage induced foci (TIF, Fig. 1B), expression of the cell-cycle checkpoint genes *p16* and *p21* (Fig. 1C) as well as genes encoding pro-inflammatory factors including IL1b and IL6 (SASP factors: Senescence-Associated Secretory Phenotype) (Fig. 1D) and hypoxia-inducible factor 1α (Fig. S1C). We also noted an elevated level of apoptosis as assayed by TUNEL (TdT-mediated dUTP Nick-End Labeling, Fig. 1E).

**Fig. 2:**
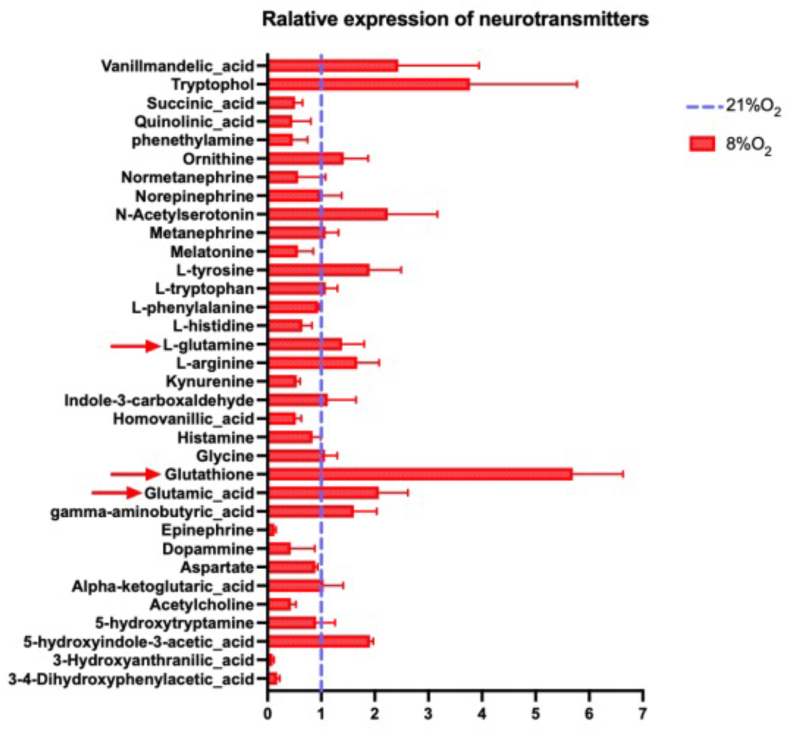
LC-MS/MS detection of neurotransmitter showed an increase of glutamatergic neurotransmitters (red arrows) upon hypoxia treatment in mouse brain which is recognized as excitotoxicity-like event (n = 3); Mann Whitney *U* test,*:p < 0.05; **:0.01<p < 0.05; ***:0.001<p < 0.01; ****:p < 0.001. (For interpretation of the references to color in this figure legend, the reader is referred to the Web version of this article.)

A metabolomic analysis of the brain (Fig. 2) revealed a significant increase of glutathione [20] and N-Acetylserotonin [18], two molecules with anti-oxidant properties, in accordance with an oxidative stress occurring in post-hypoxic brain. We also noted an increased level of the excitatory neurotransmitter glutamate, a sign of excitotoxicity previously observed upon hypoxia [2,21].

**Fig. 3:**
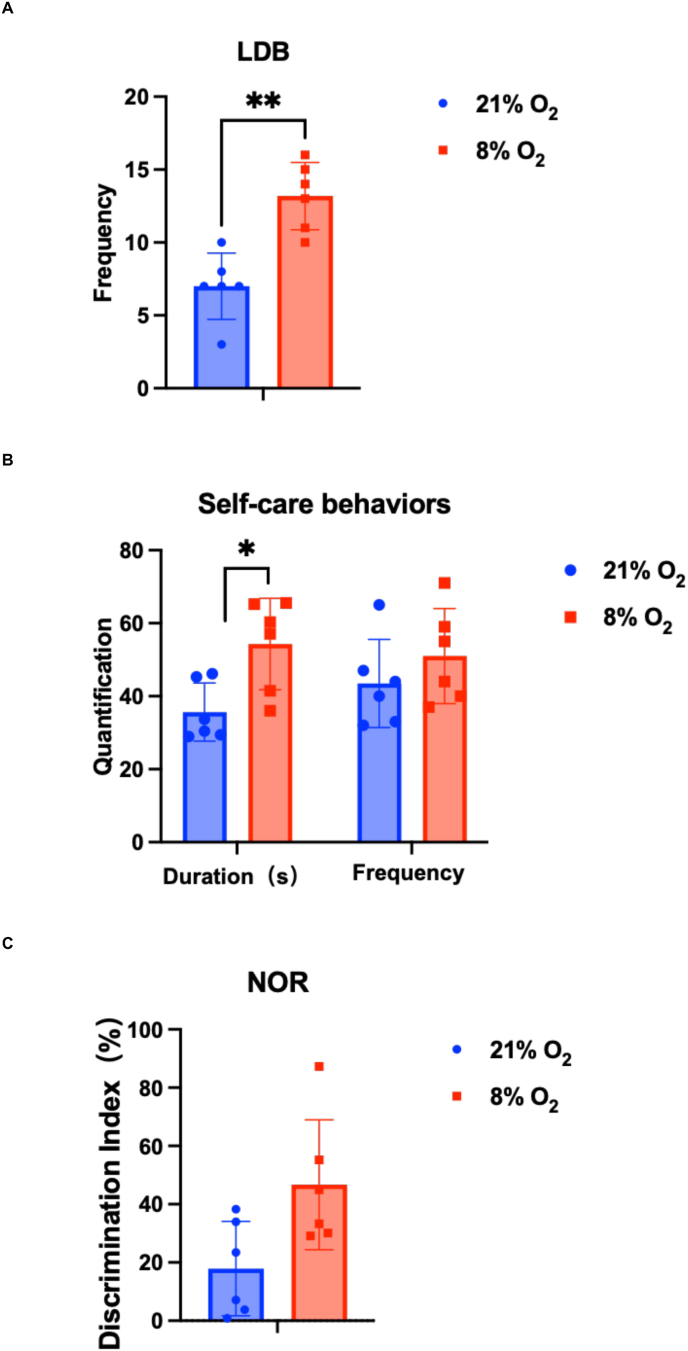
Mice under hypoxia stress showed excitotoxicity-like performance in behavioral tests. A. LDB (light/dark box) frequency showed the mice shuttled between light and dark fields. B. Latency of Self Care Behavior showed the capability of mice to adjust the environment. C. Discrimination Index of NOR (Novel Object Recognition) test evaluated the short-term memory, capacity to distinguish novel items, and the motivation to explore depending on the duration and distance of their explorations between normoxic and hypoxic group (n = 6). Paired *t*-test,*:p < 0.05; **: 0.01<p < 0.05; ***: 0.001<p < 0.01; ****: p < 0.001.

The post-hypoxic mice were also subjected to behavioral testing, including the light-dark box (LDB), new object recognition (NOR) and the self-care behavior (SCB) tests. The LBD test measures how anxious the mice are by the frequency of shuttles they make between light and dark area. The fewer the number of shuttles, the more anxious the mice are, and the more pronounced their aberrant brain activity is. The NOR test measures the amount of time mice spend examining both original and novel objects, reflecting their capacity for memory and recognition (estimated by a Discrimination Index). A low latency in the SCB test reflects the well-being of the mouse. We observed in post-hypoxic mice an increase in the frequency of light-dark field shuttles in the LDB test (Fig. 3A) and a duration of SCB (Fig. 3B). No significant change was observed in the NOR test (Fig. 3C). These behavioral changes together with the elevated level of glutamate agree with a post-hypoxic excitotoxicity effect.

### 3.2 *Terf2* downregulation contributes to the post-hypoxic brain damages

**Fig. 4:**
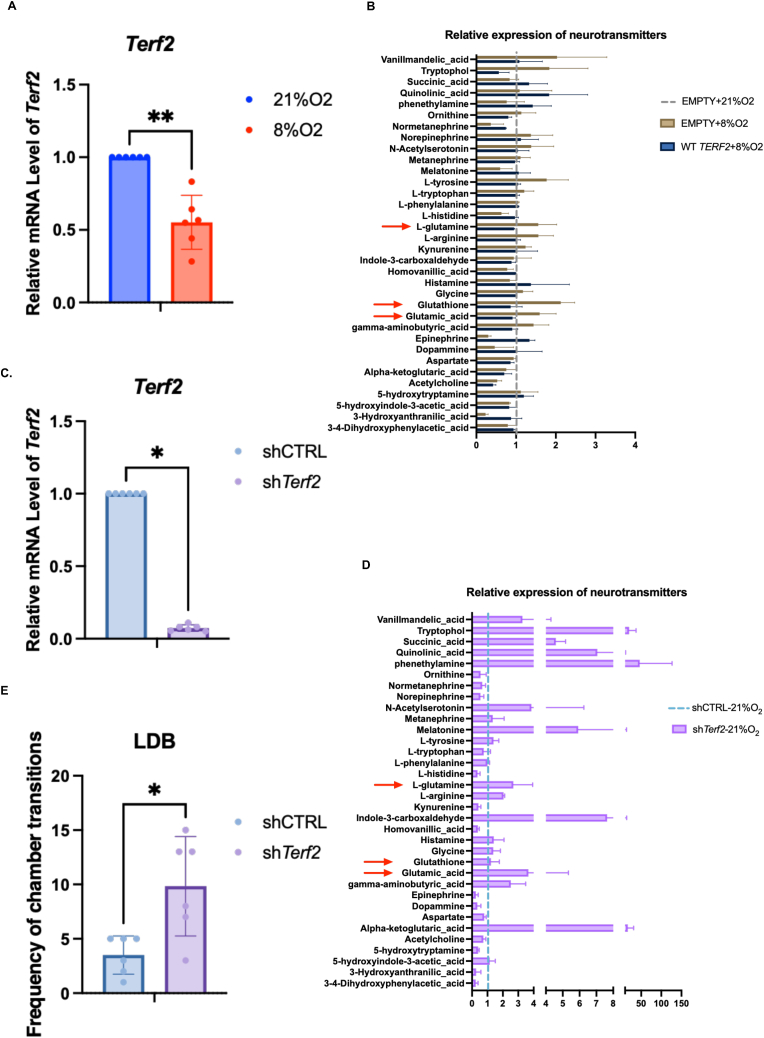
TRF2 regulates neural excitability. A. Expression of *Terf2* mRNA in the brain of hypoxic stress mice by RT-qPCR (n = 6, Mann Whitney *U* test). B: Proteomics metabolism analysis by LC-MS/MS detection of neurotransmitter in the mouse brain of hypoxic stress and overexpression of TRF2 (referred to as WT TERF2 by stereotactic injection of lentivirus to express exogenous TRF2. Red arrows indicate the neurotransmitter whose level changed due to hypoxic stress and overexpression of TRF2 (n = 3, Mann Whitney *U* test). C. Efficiency validation of *Terf2* knockdown by RT-qPCR (referred to as sh*Terf2*) after stereotactic injection of lentivirus (n = 6, Wilcoxon test). D. Proteomics metabolism analysis by LC-MS/MS detection of neurotransmitter in the mouse brain of hypoxic stress and TRF2 downregulation by stereotactic injection of sh*Terf2* lentivirus. Red arrows indicate the neurotransmitter whose level changed due to hypoxia and TRF2 knockdown (n = 3, Mann Whitney *U* test). E. Shuttles between light and dark fields of LDB test upon *Terf2* knockdown (n = 6, Paired *t*-test, *:p < 0.05; **:0.01<p < 0.05; ***:0.001<p < 0.01; ****:p < 0.001). (For interpretation of the references to color in this figure legend, the reader is referred to the Web version of this article.)

Next, we found that the *Terf2* mRNA expression in brain tissue of the post-hypoxic group was significantly lower than that of the control group (Fig. 4A). Importantly, by stereotaxic injection of lentivirus to overexpressing *Terf2* in hippocampal neural cells submitted to post-hypoxic stress, we restored a normal level of glutamate (Fig. 4B). We conclude that *Terf2* downregulation triggered by hypoxia contributes to the damaged brain phenotype.

In order to test whether a *Terf2* downregulation is sufficient to trigger an excitotoxicity effect, we microinjected a preparation of lentivirus expressing sh*Terf2* into the hippocampus region of normoxic mice (Fig. 4C). The downregulation of *Terf2* resulted in an increase in the level of glutamate (Fig. 4D). Together with an increased frequency of shuttling between bright and dark fields (Fig. 4E), this suggests that downregulation of *Terf2* in hippocampal neural cells is sufficient to trigger an excitotoxicity effect. In addition, injection of *Terf2* lentivirus leading to an overexpression of wild-type *Terf2* partially rescued the excitotoxicity-like event (Fig. S2), which is consistent with the result of the experiment in Fig. 4B.

**Fig. 5:**
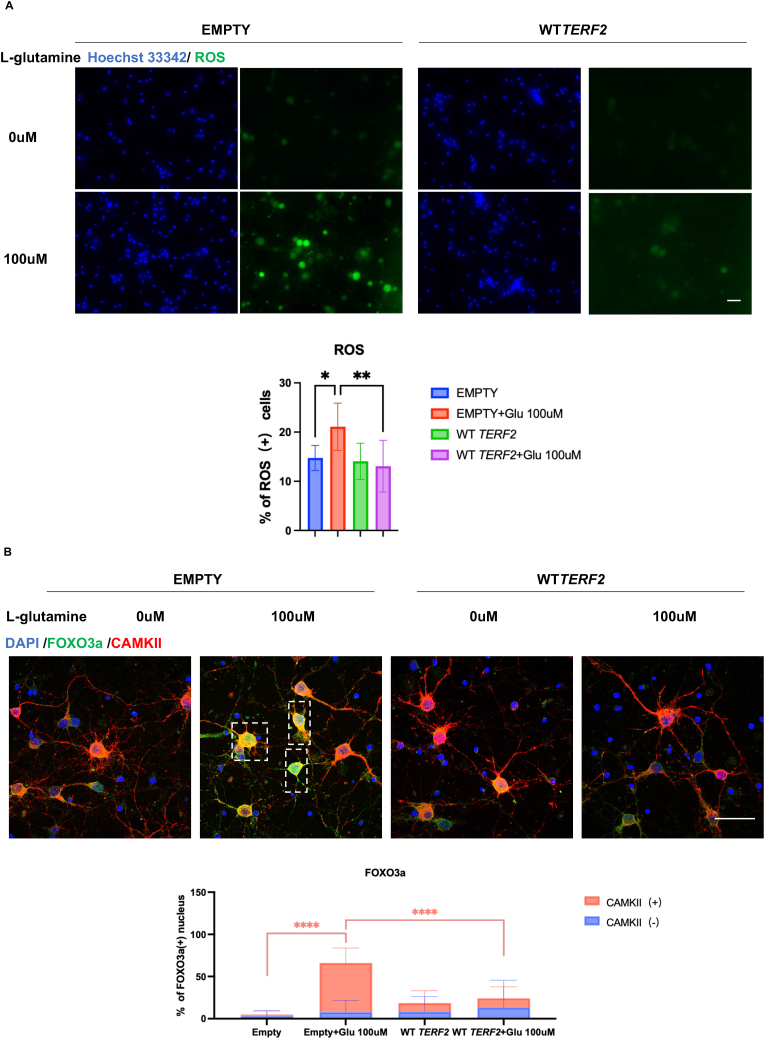
Oxidative-stress induced by glutamate is reduced under TRF2 overexpression. A. Representative images of ROS signal (green) and quantification of percentage of cells (dyed with Hoechst 33342, blue) containing ROS signal in cultured primary neuronal cells isolated from newborn mouse cortex and then transduced with lentivirus expressing exogenous TRF2 (n = 3, One Way ANOVA). B. Immunofluorescence assay and confocal imaging of FOXO3a foci (green) in primary neurons treated with l-glutamine and lentivirus transduction to overexpress TRF2 (n = 3, Two Way ANOVA). *: p < 0.05; **:0.01<p < 0.05; ***:0.001<p < 0.01; ****: p < 0.001. (For interpretation of the references to color in this figure legend, the reader is referred to the Web version of this article.)

Next, we recapitulated the pro-oxidative effects of glutamate in cultured primary neurons (Fig. 5). We found that the overexpression of *Terf2* in glutamate-treated neurons is sufficient to blunt the high level of ROS and the translocation of FOXO3A into the neuronal nucleus. This further demonstrate the protective role of TRF2 against the oxidative stress triggered by glutamate.

### 3.3 The TRF2-SIRT3 axis links post-hypoxic damage to mitochondria

**Fig. 6:**
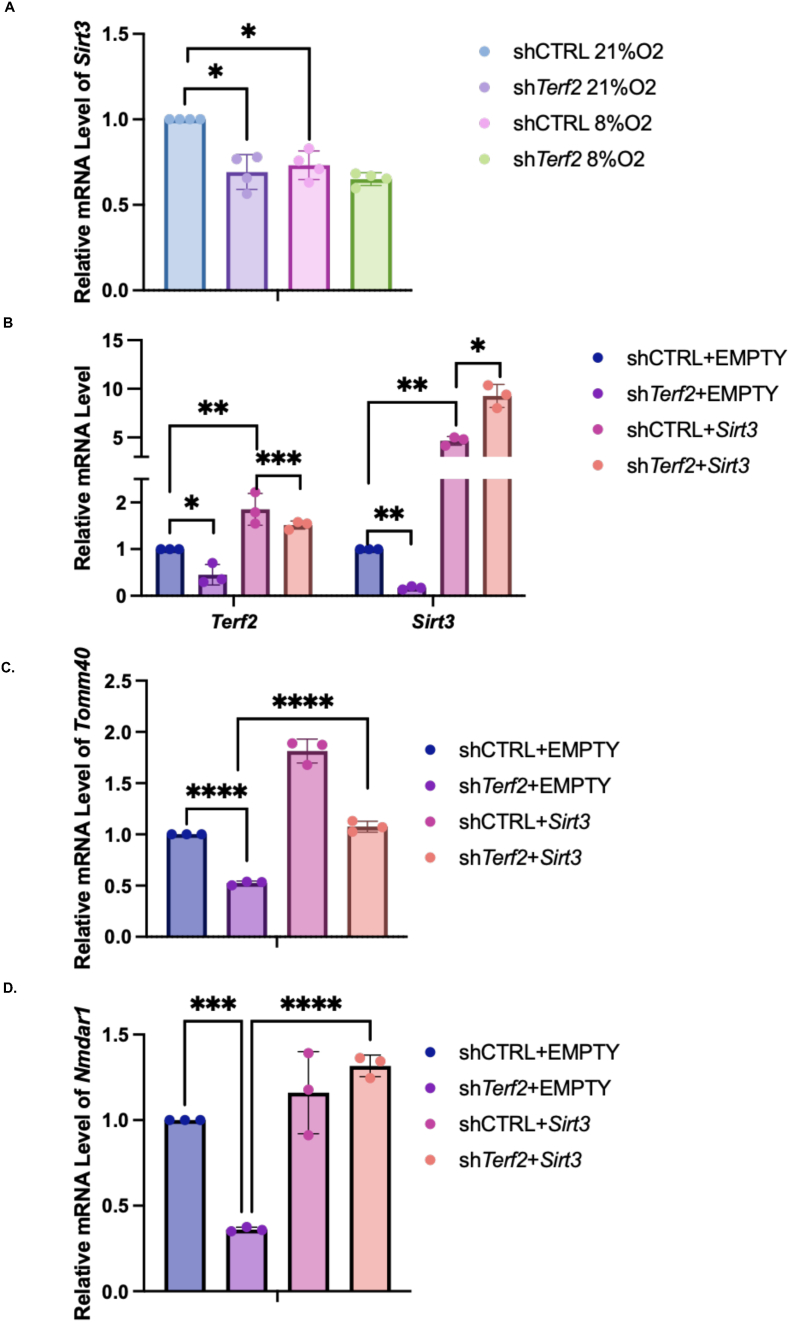
The TRF2-SIRT3 axis links post-hypoxic damage to mitochondria in primary neurons. A. RT-qPCR of *Sirt3* mRNA level of mouse brain after hypoxic treatment and lentivirus transduction of sh*Terf2* (n = 4). B. RT-qPCR of *Sirt3* and *Terft2* mRNA level in cultured primary neurons treated with lentivirus transduction of sh*Terf2* led and exogenous *Sirt3* (n = 3). C. RT-qPCR of *Sirt3* and *Terft2* mRNA level of Tomm40 in cultured primary neurons treated with lentivirus transduction of sh*Terf2* led and exogenous *Sirt3*. D. RT-qPCR of NMDA receptor 1 upon *Terf2* downregulation and *Sirt3* overexpression in cultured primary neurons (n = 3). Mann-Whitney *U* test for 6A, One Way ANOVA for 6B-D, *:p < 0.05; **:0.01<p < 0.05; ***:0.001<p < 0.01; ****:p < 0.001.

Since a functional decline of the hippocampus may be caused by mitochondrial dysfunction [22] and since, in myofibers, *Terf2* downregulation triggers mitochondrial dysfunction by repressing the expression of the mitochondrial Sirtuin *Sirt3* [8], we examined if *Sirt3* plays a role in hypoxia-triggered brain damage. Indeed, *Sirt3* mRNA expression was downregulated in the hypoxic group and upon *Terf2* downregulation in hippocampal neural cells (Fig. 6A).

Next, we tested the role of the TRF2-SIRT3 axis in cultures of primary neurons. Confirming the *in vivo* results, downregulation of *Terf2* led to repression of *Sirt3* expression (Fig. 6B). The level of mitochondrial membrane marker *Tomm40* also decreased in *Terf2*-compromised neurons (Fig. 6C). The *Tomm40* downregulation was rescued upon *Sirt3* overexpression. Finally, the expression of the receptor 1 of glutamate gene, NMDAR1, decreased upon*Terf2* mRNA downregulation, which is in agreement with an excitotoxicity effect [23], and rescued by the overexpression of *Sirt3*, (Fig. 6D).

## 4 Discussion

The main finding of this work is the involvement of the telomere protective factor TRF2 in the control of excitotoxicity triggered by post-hypoxic stress. Indeed, its expression declines in the brain of mice subjected to post-hypoxic stress and its negative regulation in the hippocampus of normoxic mice triggers an excitotoxicity effect, including an elevated level of glutamate and behavioral impairments. Importantly, by overexpressing *Terf2* in the brains of post-hypoxic stress mice, we found that glutamate content returned to normal levels. This result paves the way for a pharmacological intervention to increase the level of TRF2 in post-hypoxic stress situations to reduce neuronal damage. Our results also highlight the role played by *Sirt3,* a target of TRF2 [8], in mitochondrial dysfunction associated with post-hypoxic stress. Our results provide evidence for a TRF2-dependent link between mitochondrial control of excitotoxicity by *Sirt3*. A limit of this study is that one cannot decipher whether the effects of the modulation of *Terf2* expression in the hippocampus are due to neuronal or glial cells. Nevertheless, the fact to protect from glutamate toxicity by *Terf2* expression in cultured neurons argues that hippocampal neurons play an important role in the TRF2-dependent protection of hypoxic stress in the brain. We propose that TRF2 controls the release of glutamatergic neurotransmitters in the central nervous system by regulating the expression of *Sirt3*, opening new perspectives in the prevention and treatment of neuronal damage repair.

## Data availability statement

The datasets used and/or analyzed during the current study are available from the corresponding author on reasonable request.

## Funding

The work in the JY/YML lab was supported by the 10.13039/501100001809National Natural Science Foundation of China [grant numbers 82225018, 92149302, 81971312, 91749126, 81911530241, 81871549, 81671900]. The work in EG laboratory by the AGEMED cross-cutting program of Inserm and the ANR TELOPOST.

## CRediT authorship contribution statement

**Shuaiyun Gao:** Methodology, Investigation. **Sheng Huang:** Methodology, Software, Resources. **Yiwen Xu:** Software, Formal analysis. **Bo Wang:** Investigation, Visualization. **Peng Cheng:** Methodology, Software, Resources. **Yiming Lu:** Funding acquisition. **Eric Gilson:** Conceptualization, data interpretation and writing. **Jing Ye:** Conceptualization, data interpretation, Writing & Editing, Project administration, Funding acquisition.

## Declaration of competing interest

The authors declare that they have no known competing financial interests or personal relationships that could have appeared to influence the work reported in this paper.

## Appendix A Supplementary data

Multimedia component 1 Figure S1. Establishment of hypoxic stress model. A. Observation spot of mouse brain in this experiment. B. H&E staining of brain tissues from mice under 21% and 8% O2 (n=3). C. RT-qPCR of *Hif1* in brain from mice with hypoxic treatment (n=5). Scale bar:50um; Paired t-test, *:p<0.05; **:0.01<p<0.05;***:0.001<p<0.01;****:p<0.001. Figure S2: Shuttling analysis between light and dark fields in LDB (Light and Dark Box) assay in mice treated with 8% oxygen and lentivirus transduction to overexpress TRF2 (n=5). One Way ANOVA,*:p<0.05; **:0.01<p<0.05;***:0.001<p<0.01;****:p<0.001 Multimedia component 1
