# Supplementary material for: Role of the telomeric factor TRF2 in post-hypoxic brain damages
Source: Redox Biol. 2024 Jul 25;75:103278. doi: 10.1016/j.redox.2024.103278 (PMC11369364; doi:10.1016/j.redox.2024.103278)
Supplement: Multimedia component 1 — Figure S1. Establishment of hypoxic stress model. A. Observation spot of mouse brain in this experiment. B. H&E staining of brain tissues from mice under 21% and 8% O2 (n=3). C. RT-qPCR of Hif1 in brain from mice with hypoxic treatment (n=5). Scale bar:50um; Paired t-test, *:p<0.05; **:0.01<p<0.05;***:0.001<p<0.01;****:p<0.001. Figure S2: Shuttling analysis between light and dark fields in LDB (Light and Dark Box) assay in mice treated with 8% oxygen and lentivirus transduction to overexpress TRF2 (n=5). One Way ANOVA,*:p<0.05; **:0.01<p<0.05;***:0.001<p<0.01;****:p<0.001 [file mmc1.pdf]

Figure S1

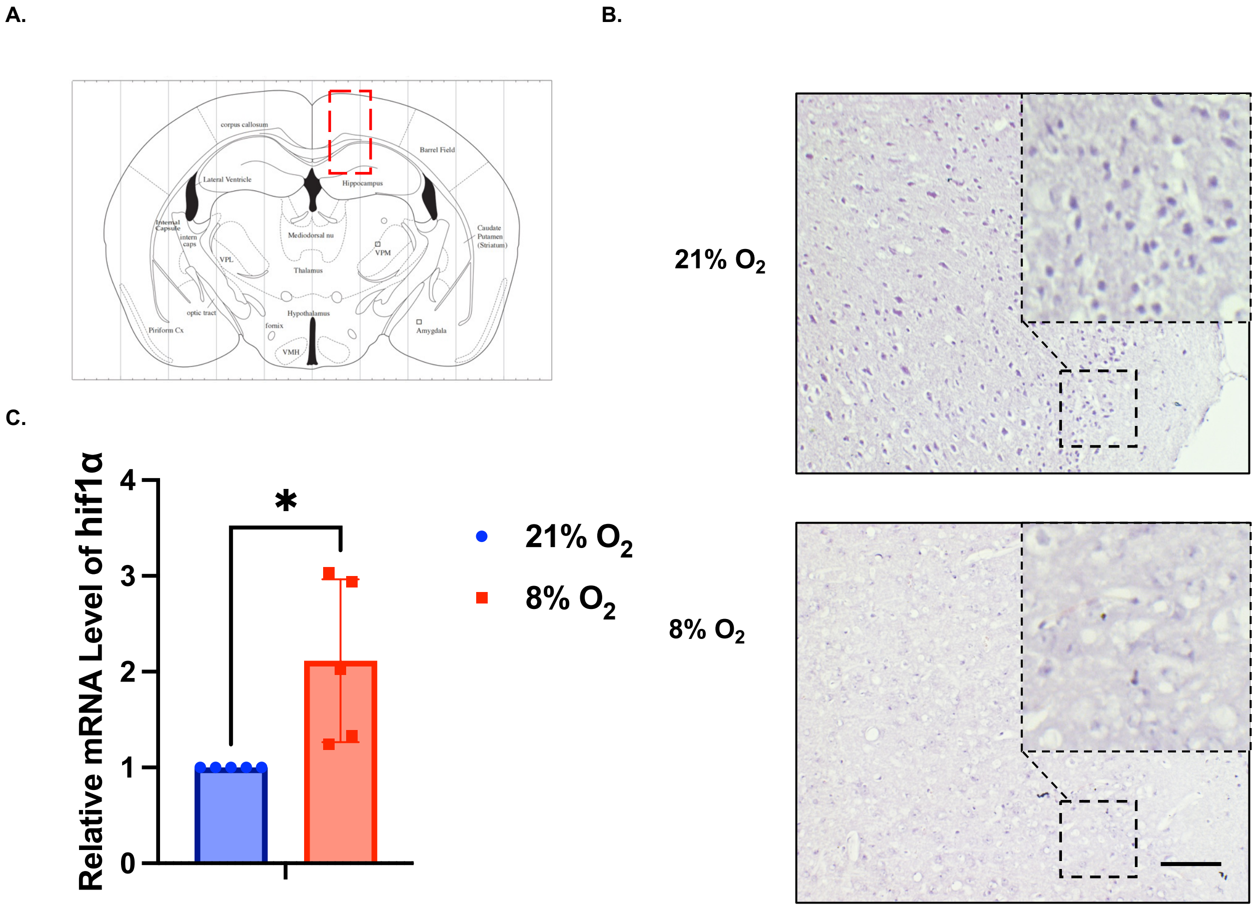

A.
B.
corpus callosum
Barrel Field
Lateral Ventricle
Hippocampus
Internal Capsule
intern caps
Mediodorsal nu
Caudate Putamen (Striatum)
VPL
VPM
Thalamus
optic tract
Hypothalamus
Piriform Cx
fornix
Amygdala
VMH
21% O2
8% O2
C.
Relative mRNA Level of hif1α
4
3
2
1
0
*
21% O2
8% O2

Figure S2

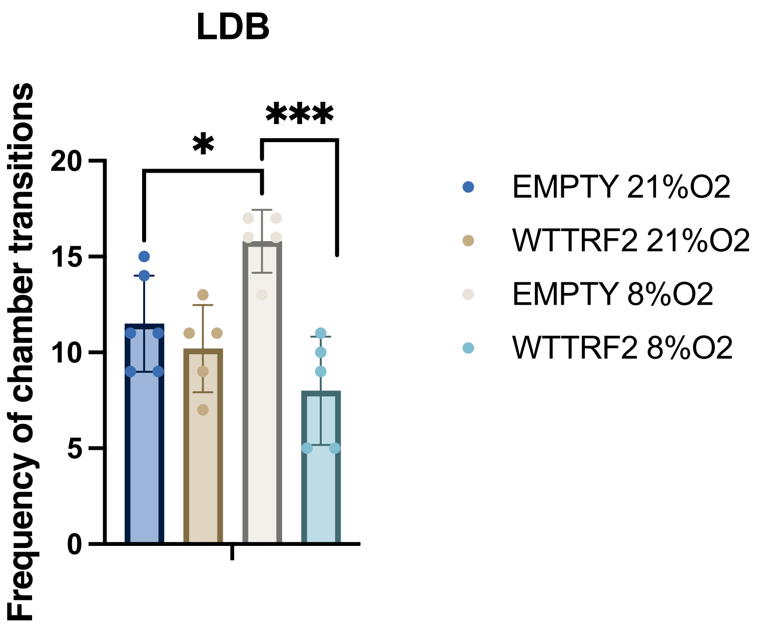
LDB
Frequency of chamber transitions
20
15
10
5
0
*
***
EMPTY 21%O2
WTTRF2 21%O2
EMPTY 8%O2
WTTRF2 8%O2

Table S1

Table S1. Solutions in FISH

**Wash I (pH=7.2)**	Formaldehyde	70ml
	1M Tris-HCl（pH=7.4）	1ml
	H_2O	29ml
**Wash II（pH=7.5)**	10% Tween-20	10ml
	1M Tris-HCl（pH=7.4）	1ml
	5M NaCl	6ml
	H_2O	183ml
**Probe Buffer**	10% blocking buffer	5ul
	Probe	1ul
	Wash I	To 100ul

Table S2

Table S2. Information of primers used.

Primer	Sequence(5'-3')
Mouse-tomm40	F: GGGAGTCCAACTACCACTTTG
	R: CTCAATGCACAGGTCATCCA
Mouse-hif1a	F: TCTCGGCGAAGCAAAGAGTC
	R:AGCCATCTAGGGCTTTCAGATAA
Mouse-gfap	F: ACCAAACTGGCTGATGTCTA
	R: CTCCAGCGATTCAACCTTTC
Mouse-p16	F: GCTCAACTACGGTGCAGATTC
	R: GCACGATGTCTTGATGTCC
Mouse- p21	F: CCTGGTGATGTCCGACCTG
	R: CCATGAGCGCATCGCAATC
Mouse-il1β	F: GGTACATCAGCACCTCACAA
	R: TTAGAAACAGTCCAGCCCATAC
Mouse-il6	F: CTTCCATCCAGTTGCCTTCT
	R: CTATACCACTTCACAAGTCGGAG
Mouse-sirt3	F: GTTCTGAGTCCTCGAAGGAAAG
	R: AGATCCAGCAGTTCTTGTGTC
Mouse-dcx	F: AGGGAGTGCGCTACATTTAT
	R: CTGCGAATGATGGTCACAAG

Table S3

Table S3. Information of antibodies used.

Antibody	Origin	Company	CAT. Number
**Anti-53BP1 antibody**	Rabbit	CST	4937s
**Anti-TRF2 antibody**	Rabbit	Novus	NB-57130
**Anti-GAPDH antibody**	Rabbit	Yeasen	30202ES60
**Anti-NeuN antibody**	Mouse	Millipore	MAB377

Table S4

Table S4. Information of lentivirus.

Lentivirus	Neucleotide
wtTRF2	NM_009353
wtSIRT3	NM_022433.2

Lentivirus	Sequence(5'-3')
shTRF2	CCCTTGGAATCAGCTATCAAT
